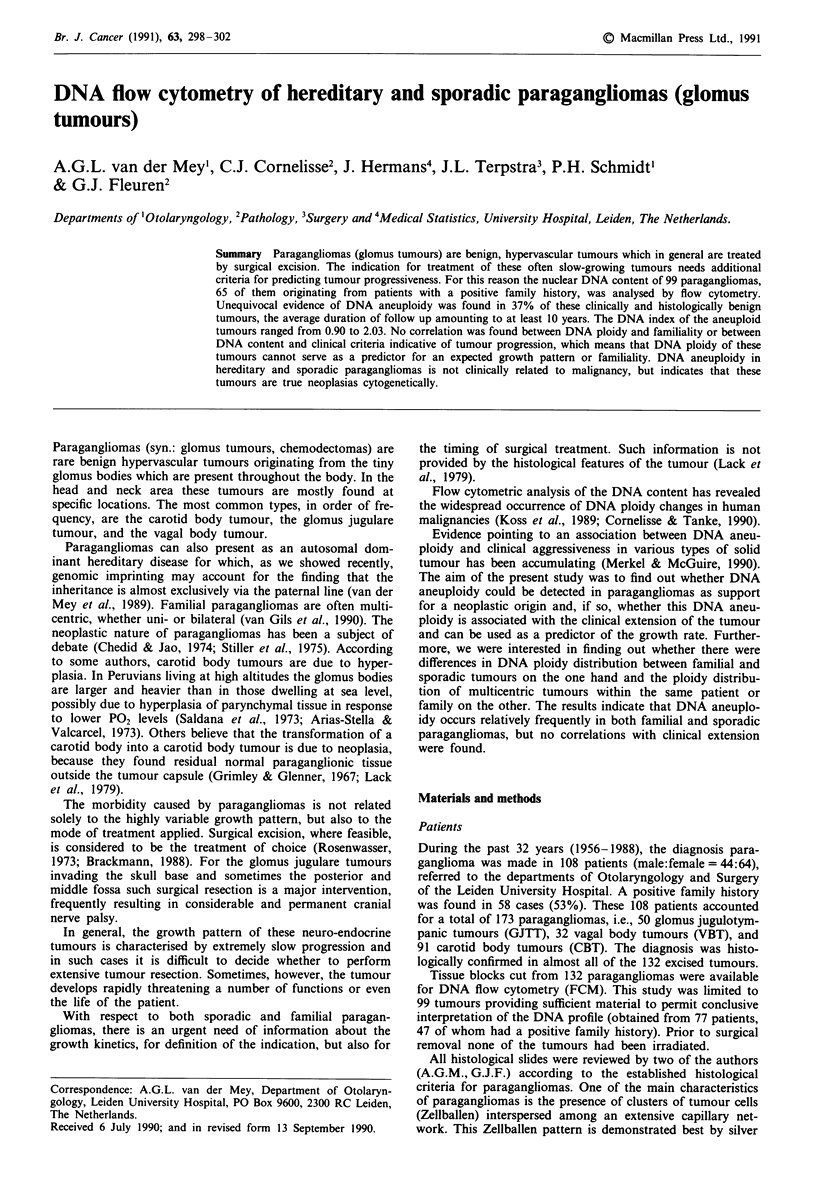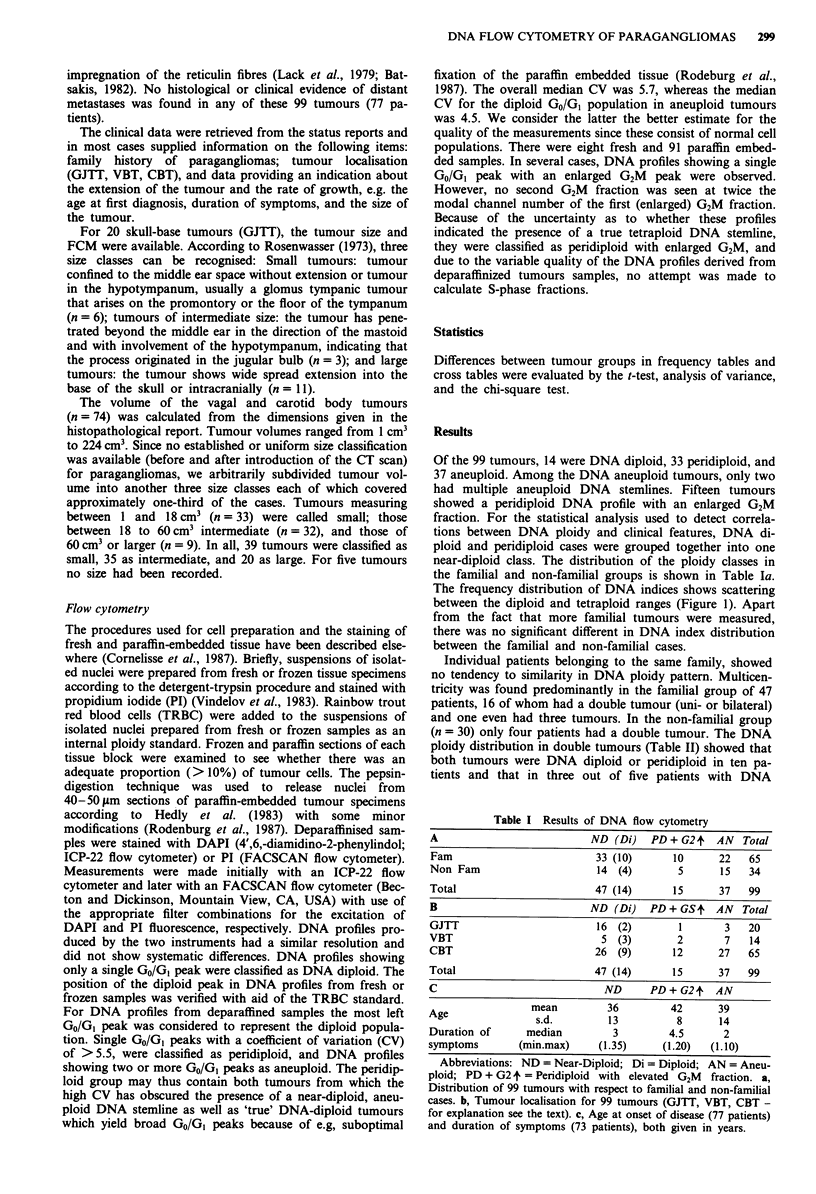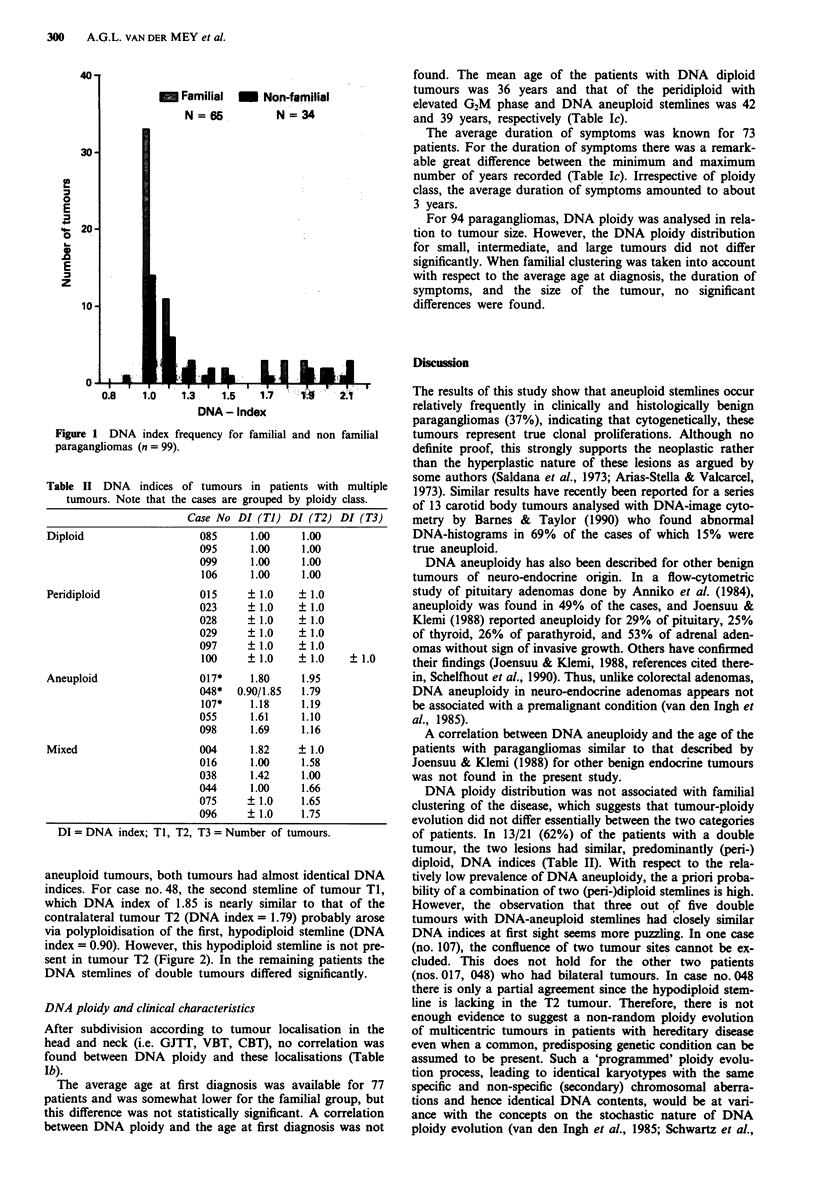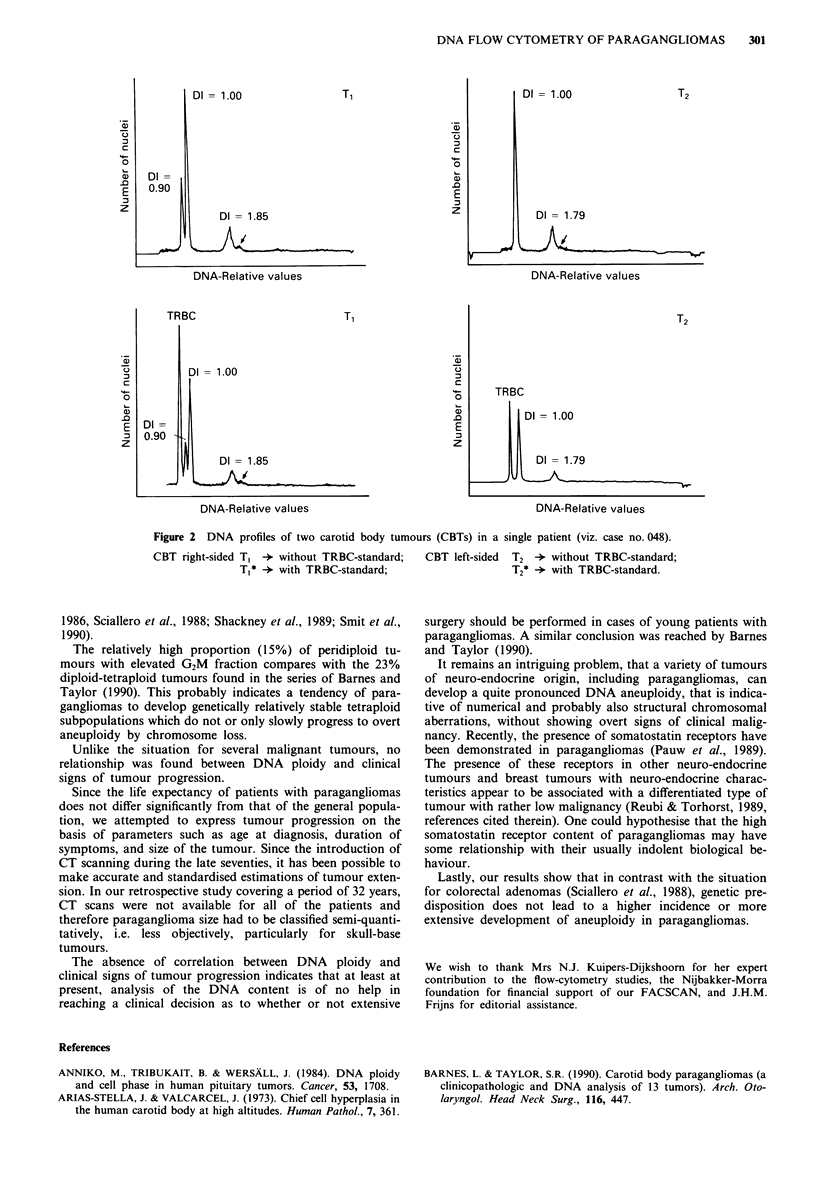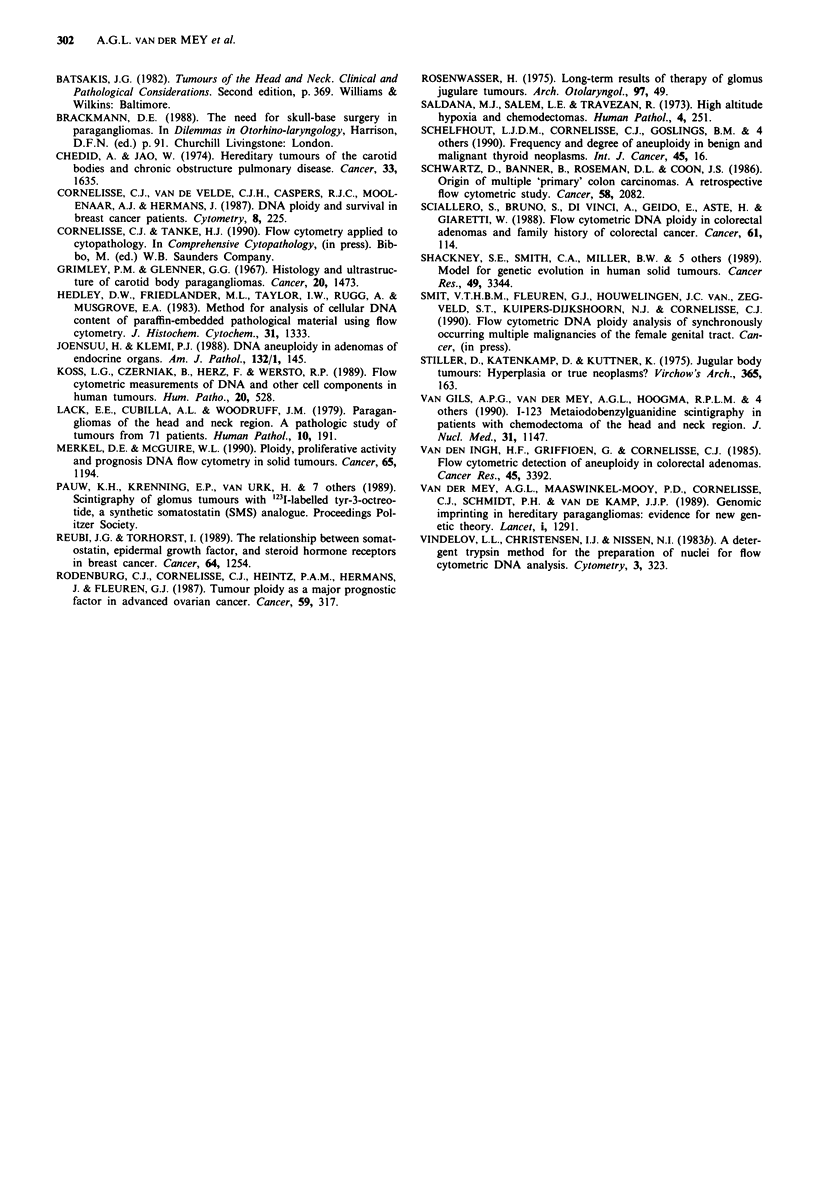# DNA flow cytometry of hereditary and sporadic paragangliomas (glomus tumours).

**DOI:** 10.1038/bjc.1991.69

**Published:** 1991-02

**Authors:** A. G. van der Mey, C. J. Cornelisse, J. Hermans, J. L. Terpstra, P. H. Schmidt, G. J. Fleuren

**Affiliations:** Department of Otolaryngology, University Hospital, Leiden, The Netherlands.

## Abstract

Paragangliomas (glomus tumours) are benign, hypervascular tumours which in general are treated by surgical excision. The indication for treatment of these often slow-growing tumours needs additional criteria for predicting tumour progressiveness. For this reason the nuclear DNA content of 99 paragangliomas, 65 of them originating from patients with a positive family history, was analysed by flow cytometry. Unequivocal evidence of DNA aneuploidy was found in 37% of these clinically and histologically benign tumours, the average duration of follow up amounting to at least 10 years. The DNA index of the aneuploid tumours ranged from 0.90 to 2.03. No correlation was found between DNA ploidy and familiality or between DNA content and clinical criteria indicative of tumour progression, which means that DNA ploidy of these tumours cannot serve as a predictor for an expected growth pattern or familiality. DNA aneuploidy in hereditary and sporadic paragangliomas is not clinically related to malignancy, but indicates that these tumours are true neoplasias cytogenetically.


Br. J. Cancer (1991), 63, 298-302 © Macmillan Press Ltd., 1991

### DNA flow cytometry of hereditary and sporadic paragangliomas (glomus tumours)

A.G.L. van der Mey[1], C.J. Cornelisse[2], J. Hermans[4], J.L. Terpstra[3], P.H. Schmidt[1] & G.J. Fleuren[2]

*Departments of [1]Otolaryngology, [2]Pathology, [3]Surgery and [4]Medical Statistics, University Hospital, Leiden, The Netherlands.*

**Summary** Paragangliomas (glomus tumours) are benign, hypervascular tumours which in general are treated by surgical excision. The indication for treatment of these often slow-growing tumours needs additional criteria for predicting tumour progressiveness. For this reason the nuclear DNA content of 99 paragangliomas, 65 of them originating from patients with a positive family history, was analysed by flow cytometry. Unequivocal evidence of DNA aneuploidy was found in 37% of these clinically and histologically benign tumours, the average duration of follow up amounting to at least 10 years. The DNA index of the aneuploid tumours ranged from 0.90 to 2.03. No correlation was found between DNA ploidy and familiality or between DNA content and clinical criteria indicative of tumour progression, which means that DNA ploidy of these tumours cannot serve as a predictor for an expected growth pattern or familiality. DNA aneuploidy in hereditary and sporadic paragangliomas is not clinically related to malignancy, but indicates that these tumours are true neoplasias cytogenetically.

Paragangliomas (syn.: glomus tumours, chemodectomas) are rare benign hypervascular tumours originating from the tiny glomus bodies which are present throughout the body. In the head and neck area these tumours are mostly found at specific locations. The most common types, in order of frequency, are the carotid body tumour, the glomus jugulare tumour, and the vagal body tumour.

Paragangliomas can also present as an autosomal dominant hereditary disease for which, as we showed recently, genomic imprinting may account for the finding that the inheritance is almost exclusively via the paternal line (van der Mey *et al.*, 1989). Familial paragangliomas are often multicentric, whether uni- or bilateral (van Gils *et al.*, 1990). The neoplastic nature of paragangliomas has been a subject of debate (Chedid & Jao, 1974; Stiller *et al.*, 1975). According to some authors, carotid body tumours are due to hyperplasia. In Peruvians living at high altitudes the glomus bodies are larger and heavier than in those dwelling at sea level, possibly due to hyperplasia of parynchymal tissue in response to lower $PO_2$ levels (Saldana *et al.*, 1973; Arias-Stella & Valcarcel, 1973). Others believe that the transformation of a carotid body into a carotid body tumour is due to neoplasia, because they found residual normal paraganglionic tissue outside the tumour capsule (Grimley & Glenner, 1967; Lack *et al.*, 1979).

The morbidity caused by paragangliomas is not related solely to the highly variable growth pattern, but also to the mode of treatment applied. Surgical excision, where feasible, is considered to be the treatment of choice (Rosenwasser, 1973; Brackmann, 1988). For the glomus jugulare tumours invading the skull base and sometimes the posterior and middle fossa such surgical resection is a major intervention, frequently resulting in considerable and permanent cranial nerve palsy.

In general, the growth pattern of these neuro-endocrine tumours is characterised by extremely slow progression and in such cases it is difficult to decide whether to perform extensive tumour resection. Sometimes, however, the tumour develops rapidly threatening a number of functions or even the life of the patient.

With respect to both sporadic and familial paragangliomas, there is an urgent need of information about the growth kinetics, for definition of the indication, but also for the timing of surgical treatment. Such information is not provided by the histological features of the tumour (Lack *et al.*, 1979).

Flow cytometric analysis of the DNA content has revealed the widespread occurrence of DNA ploidy changes in human malignancies (Koss *et al.*, 1989; Cornelisse & Tanke, 1990).

Evidence pointing to an association between DNA aneuploidy and clinical aggressiveness in various types of solid tumour has been accumulating (Merkel & McGuire, 1990). The aim of the present study was to find out whether DNA aneuploidy could be detected in paragangliomas as support for a neoplastic origin and, if so, whether this DNA aneuploidy is associated with the clinical extension of the tumour and can be used as a predictor of the growth rate. Furthermore, we were interested in finding out whether there were differences in DNA ploidy distribution between familial and sporadic tumours on the one hand and the ploidy distribution of multicentric tumours within the same patient or family on the other. The results indicate that DNA aneuploidy occurs relatively frequently in both familial and sporadic paragangliomas, but no correlations with clinical extension were found.

### Materials and methods

### Patients

During the past 32 years (1956-1988), the diagnosis paraganglioma was made in 108 patients (male:female = 44:64), referred to the departments of Otolaryngology and Surgery of the Leiden University Hospital. A positive family history was found in 58 cases (53%). These 108 patients accounted for a total of 173 paragangliomas, i.e., 50 glomus jugulotympanic tumours (GJTT), 32 vagal body tumours (VBT), and 91 carotid body tumours (CBT). The diagnosis was histologically confirmed in almost all of the 132 excised tumours.

Tissue blocks cut from 132 paragangliomas were available for DNA flow cytometry (FCM). This study was limited to 99 tumours providing sufficient material to permit conclusive interpretation of the DNA profile (obtained from 77 patients, 47 of whom had a positive family history). Prior to surgical removal none of the tumours had been irradiated.

All histological slides were reviewed by two of the authors (A.G.M., G.J.F.) according to the established histological criteria for paragangliomas. One of the main characteristics of paragangliomas is the presence of clusters of tumour cells (Zellballen) interspersed among an extensive capillary network. This Zellballen pattern is demonstrated best by silver

Correspondence: A.G.L. van der Mey, Department of Otolaryngology, Leiden University Hospital, PO Box 9600, 2300 RC Leiden, The Netherlands.
Received 6 July 1990; and in revised form 13 September 1990.

impregnation of the reticulin fibres (Lack *et al.*, 1979; Batsakis, 1982). No histological or clinical evidence of distant metastases was found in any of these 99 tumours (77 patients).

The clinical data were retrieved from the status reports and in most cases supplied information on the following items: family history of paragangliomas; tumour localisation (GJTT, VBT, CBT), and data providing an indication about the extension of the tumour and the rate of growth, e.g. the age at first diagnosis, duration of symptoms, and the size of the tumour.

For 20 skull-base tumours (GJTT), the tumour size and FCM were available. According to Rosenwasser (1973), three size classes can be recognised: Small tumours: tumour confined to the middle ear space without extension or tumour in the hypotympanum, usually a glomus tympanic tumour that arises on the promontory or the floor of the tympanum ($n = 6$); tumours of intermediate size: the tumour has penetrated beyond the middle ear in the direction of the mastoid and with involvement of the hypotympanum, indicating that the process originated in the jugular bulb ($n = 3$); and large tumours: the tumour shows wide spread extension into the base of the skull or intracranially ($n = 11$).

The volume of the vagal and carotid body tumours ($n = 74$) was calculated from the dimensions given in the histopathological report. Tumour volumes ranged from 1 $cm^3$ to 224 $cm^3$. Since no established or uniform size classification was available (before and after introduction of the CT scan) for paragangliomas, we arbitrarily subdivided tumour volume into another three size classes each of which covered approximately one-third of the cases. Tumours measuring between 1 and 18 $cm^3$ ($n = 33$) were called small; those between 18 to 60 $cm^3$ intermediate ($n = 32$), and those of 60 $cm^3$ or larger ($n = 9$). In all, 39 tumours were classified as small, 35 as intermediate, and 20 as large. For five tumours no size had been recorded.

### *Flow cytometry*

The procedures used for cell preparation and the staining of fresh and paraffin-embedded tissue have been described elsewhere (Cornelisse *et al.*, 1987). Briefly, suspensions of isolated nuclei were prepared from fresh or frozen tissue specimens according to the detergent-trypsin procedure and stained with propidium iodide (PI) (Vindelov *et al.*, 1983). Rainbow trout red blood cells (TRBC) were added to the suspensions of isolated nuclei prepared from fresh or frozen samples as an internal ploidy standard. Frozen and paraffin sections of each tissue block were examined to see whether there was an adequate proportion ($>10\%$) of tumour cells. The pepsin-digestion technique was used to release nuclei from 40–50 $\mu m$ sections of paraffin-embedded tumour specimens according to Hedly *et al.* (1983) with some minor modifications (Rodenburg *et al.*, 1987). Deparaffinised samples were stained with DAPI (4′,6,-diamidino-2-phenylindol; ICP-22 flow cytometer) or PI (FACSCAN flow cytometer). Measurements were made initially with an ICP-22 flow cytometer and later with an FACSCAN flow cytometer (Becton and Dickinson, Mountain View, CA, USA) with use of the appropriate filter combinations for the excitation of DAPI and PI fluorescence, respectively. DNA profiles produced by the two instruments had a similar resolution and did not show systematic differences. DNA profiles showing only a single $G_0/G_1$ peak were classified as DNA diploid. The position of the diploid peak in DNA profiles from fresh or frozen samples was verified with aid of the TRBC standard. For DNA profiles from deparaffined samples the most left $G_0/G_1$ peak was considered to represent the diploid population. Single $G_0/G_1$ peaks with a coefficient of variation (CV) of $>5.5$, were classified as peridiploid, and DNA profiles showing two or more $G_0/G_1$ peaks as aneuploid. The peridiploid group may thus contain both tumours from which the high CV has obscured the presence of a near-diploid, aneuploid DNA stemline as well as 'true' DNA-diploid tumours which yield broad $G_0/G_1$ peaks because of e.g, suboptimal fixation of the paraffin embedded tissue (Rodeburg *et al.*, 1987). The overall median CV was 5.7, whereas the median CV for the diploid $G_0/G_1$ population in aneuploid tumours was 4.5. We consider the latter the better estimate for the quality of the measurements since these consist of normal cell populations. There were eight fresh and 91 paraffin embedded samples. In several cases, DNA profiles showing a single $G_0/G_1$ peak with an enlarged $G_2M$ peak were observed. However, no second $G_2M$ fraction was seen at twice the modal channel number of the first (enlarged) $G_2M$ fraction. Because of the uncertainty as to whether these profiles indicated the presence of a true tetraploid DNA stemline, they were classified as peridiploid with enlarged $G_2M$, and due to the variable quality of the DNA profiles derived from deparaffinized tumours samples, no attempt was made to calculate S-phase fractions.

### Statistics

Differences between tumour groups in frequency tables and cross tables were evaluated by the *t*-test, analysis of variance, and the chi-square test.

### Results

Of the 99 tumours, 14 were DNA diploid, 33 peridiploid, and 37 aneuploid. Among the DNA aneuploid tumours, only two had multiple aneuploid DNA stemlines. Fifteen tumours showed a peridiploid DNA profile with an enlarged $G_2M$ fraction. For the statistical analysis used to detect correlations between DNA ploidy and clinical features, DNA diploid and peridiploid cases were grouped together into one near-diploid class. The distribution of the ploidy classes in the familial and non-familial groups is shown in Table I*a*. The frequency distribution of DNA indices shows scattering between the diploid and tetraploid ranges (Figure 1). Apart from the fact that more familial tumours were measured, there was no significant different in DNA index distribution between the familial and non-familial cases.

Individual patients belonging to the same family, showed no tendency to similarity in DNA ploidy pattern. Multicentricity was found predominantly in the familial group of 47 patients, 16 of whom had a double tumour (uni- or bilateral) and one even had three tumours. In the non-familial group ($n = 30$) only four patients had a double tumour. The DNA ploidy distribution in double tumours (Table II) showed that both tumours were DNA diploid or peridiploid in ten patients and that in three out of five patients with DNA

**Table I** Results of DNA flow cytometry

| A | | *ND (Di)* | *PD + G2↑* | *AN* | *Total* |
|---|---|---|---|---|---|
| Fam | | 33 (10) | 10 | 22 | 65 |
| Non Fam | | 14 (4) | 5 | 15 | 34 |
| Total | | 47 (14) | 15 | 37 | 99 |
| **B** | | *ND (Di)* | *PD + GS↑* | *AN* | *Total* |
| GJTT | | 16 (2) | 1 | 3 | 20 |
| VBT | | 5 (3) | 2 | 7 | 14 |
| CBT | | 26 (9) | 12 | 27 | 65 |
| Total | | 47 (14) | 15 | 37 | 99 |
| **C** | | *ND* | *PD + G2↑* | *AN* | |
| Age | mean | 36 | 42 | 39 | |
| | s.d. | 13 | 8 | 14 | |
| Duration of symptoms | median (min.max) | 3 (1.35) | 4.5 (1.20) | 2 (1.10) | |

Abbreviations: ND = Near-Diploid; Di = Diploid; AN = Aneuploid; PD + G2↑ = Peridiploid with elevated $G_2M$ fraction. **a**, Distribution of 99 tumours with respect to familial and non-familial cases. **b**, Tumour localisation for 99 tumours (GJTT, VBT, CBT – for explanation see the text). **c**, Age at onset of disease (77 patients) and duration of symptoms (73 patients), both given in years.

**Figure 1** DNA index frequency for familial and non familial paragangliomas ($n = 99$).

**Table II** DNA indices of tumours in patients with multiple tumours. Note that the cases are grouped by ploidy class.

| | *Case No* | *DI (T1)* | *DI (T2)* | *DI (T3)* |
|---|---|---|---|---|
| Diploid | 085 | 1.00 | 1.00 | |
| | 095 | 1.00 | 1.00 | |
| | 099 | 1.00 | 1.00 | |
| | 106 | 1.00 | 1.00 | |
| Peridiploid | 015 | ± 1.0 | ± 1.0 | |
| | 023 | ± 1.0 | ± 1.0 | |
| | 028 | ± 1.0 | ± 1.0 | |
| | 029 | ± 1.0 | ± 1.0 | |
| | 097 | ± 1.0 | ± 1.0 | |
| | 100 | ± 1.0 | ± 1.0 | ± 1.0 |
| Aneuploid | 017* | 1.80 | 1.95 | |
| | 048* | 0.90/1.85 | 1.79 | |
| | 107* | 1.18 | 1.19 | |
| | 055 | 1.61 | 1.10 | |
| | 098 | 1.69 | 1.16 | |
| Mixed | 004 | 1.82 | ± 1.0 | |
| | 016 | 1.00 | 1.58 | |
| | 038 | 1.42 | 1.00 | |
| | 044 | 1.00 | 1.66 | |
| | 075 | ± 1.0 | 1.65 | |
| | 096 | ± 1.0 | 1.75 | |

DI = DNA index; T1, T2, T3 = Number of tumours.

aneuploid tumours, both tumours had almost identical DNA indices. For case no. 48, the second stemline of tumour T1, which DNA index of 1.85 is nearly similar to that of the contralateral tumour T2 (DNA index = 1.79) probably arose via polyploidisation of the first, hypodiploid stemline (DNA index = 0.90). However, this hypodiploid stemline is not present in tumour T2 (Figure 2). In the remaining patients the DNA stemlines of double tumours differed significantly.

### *DNA ploidy and clinical characteristics*

After subdivision according to tumour localisation in the head and neck (i.e. GJTT, VBT, CBT), no correlation was found between DNA ploidy and these localisations (Table I*b*).

The average age at first diagnosis was available for 77 patients and was somewhat lower for the familial group, but this difference was not statistically significant. A correlation between DNA ploidy and the age at first diagnosis was not found. The mean age of the patients with DNA diploid tumours was 36 years and that of the peridiploid with elevated $G_2M$ phase and DNA aneuploid stemlines was 42 and 39 years, respectively (Table I*c*).

The average duration of symptoms was known for 73 patients. For the duration of symptoms there was a remarkable great difference between the minimum and maximum number of years recorded (Table I*c*). Irrespective of ploidy class, the average duration of symptoms amounted to about 3 years.

For 94 paragangliomas, DNA ploidy was analysed in relation to tumour size. However, the DNA ploidy distribution for small, intermediate, and large tumours did not differ significantly. When familial clustering was taken into account with respect to the average age at diagnosis, the duration of symptoms, and the size of the tumour, no significant differences were found.

### Discussion

The results of this study show that aneuploid stemlines occur relatively frequently in clinically and histologically benign paragangliomas (37%), indicating that cytogenetically, these tumours represent true clonal proliferations. Although no definite proof, this strongly supports the neoplastic rather than the hyperplastic nature of these lesions as argued by some authors (Saldana *et al.*, 1973; Arias-Stella & Valcarcel, 1973). Similar results have recently been reported for a series of 13 carotid body tumours analysed with DNA-image cytometry by Barnes & Taylor (1990) who found abnormal DNA-histograms in 69% of the cases of which 15% were true aneuploid.

DNA aneuploidy has also been described for other benign tumours of neuro-endocrine origin. In a flow-cytometric study of pituitary adenomas done by Anniko *et al.* (1984), aneuploidy was found in 49% of the cases, and Joensuu & Klemi (1988) reported aneuploidy for 29% of pituitary, 25% of thyroid, 26% of parathyroid, and 53% of adrenal adenomas without sign of invasive growth. Others have confirmed their findings (Joensuu & Klemi, 1988, references cited therein, Schelfhout *et al.*, 1990). Thus, unlike colorectal adenomas, DNA aneuploidy in neuro-endocrine adenomas appears not be associated with a premalignant condition (van den Ingh *et al.*, 1985).

A correlation between DNA aneuploidy and the age of the patients with paragangliomas similar to that described by Joensuu & Klemi (1988) for other benign endocrine tumours was not found in the present study.

DNA ploidy distribution was not associated with familial clustering of the disease, which suggests that tumour-ploidy evolution did not differ essentially between the two categories of patients. In 13/21 (62%) of the patients with a double tumour, the two lesions had similar, predominantly (peri-) diploid, DNA indices (Table II). With respect to the relatively low prevalence of DNA aneuploidy, the a priori probability of a combination of two (peri-)diploid stemlines is high. However, the observation that three out of five double tumours with DNA-aneuploid stemlines had closely similar DNA indices at first sight seems more puzzling. In one case (no. 107), the confluence of two tumour sites cannot be excluded. This does not hold for the other two patients (nos. 017, 048) who had bilateral tumours. In case no. 048 there is only a partial agreement since the hypodiploid stemline is lacking in the T2 tumour. Therefore, there is not enough evidence to suggest a non-random ploidy evolution of multicentric tumours in patients with hereditary disease even when a common, predisposing genetic condition can be assumed to be present. Such a 'programmed' ploidy evolution process, leading to identical karyotypes with the same specific and non-specific (secondary) chromosomal aberrations and hence identical DNA contents, would be at variance with the concepts on the stochastic nature of DNA ploidy evolution (van den Ingh *et al.*, 1985; Schwartz *et al.*,

**Figure 2** DNA profiles of two carotid body tumours (CBTs) in a single patient (viz. case no. 048).
CBT right-sided $T_1$ → without TRBC-standard; $T_1$* → with TRBC-standard; CBT left-sided $T_2$ → without TRBC-standard; $T_2$* → with TRBC-standard.

1986, Sciallero *et al.*, 1988; Shackney *et al.*, 1989; Smit *et al.*, 1990).

The relatively high proportion (15%) of peridiploid tumours with elevated $G_2M$ fraction compares with the 23% diploid-tetraploid tumours found in the series of Barnes and Taylor (1990). This probably indicates a tendency of paragangliomas to develop genetically relatively stable tetraploid subpopulations which do not or only slowly progress to overt aneuploidy by chromosome loss.

Unlike the situation for several malignant tumours, no relationship was found between DNA ploidy and clinical signs of tumour progression.

Since the life expectancy of patients with paragangliomas does not differ significantly from that of the general population, we attempted to express tumour progression on the basis of parameters such as age at diagnosis, duration of symptoms, and size of the tumour. Since the introduction of CT scanning during the late seventies, it has been possible to make accurate and standardised estimations of tumour extension. In our retrospective study covering a period of 32 years, CT scans were not available for all of the patients and therefore paraganglioma size had to be classified semi-quantitatively, i.e. less objectively, particularly for skull-base tumours.

The absence of correlation between DNA ploidy and clinical signs of tumour progression indicates that at least at present, analysis of the DNA content is of no help in reaching a clinical decision as to whether or not extensive surgery should be performed in cases of young patients with paragangliomas. A similar conclusion was reached by Barnes and Taylor (1990).

It remains an intriguing problem, that a variety of tumours of neuro-endocrine origin, including paragangliomas, can develop a quite pronounced DNA aneuploidy, that is indicative of numerical and probably also structural chromosomal aberrations, without showing overt signs of clinical malignancy. Recently, the presence of somatostatin receptors have been demonstrated in paragangliomas (Pauw *et al.*, 1989). The presence of these receptors in other neuro-endocrine tumours and breast tumours with neuro-endocrine characteristics appear to be associated with a differentiated type of tumour with rather low malignancy (Reubi & Torhorst, 1989, references cited therein). One could hypothesise that the high somatostatin receptor content of paragangliomas may have some relationship with their usually indolent biological behaviour.

Lastly, our results show that in contrast with the situation for colorectal adenomas (Sciallero *et al.*, 1988), genetic predisposition does not lead to a higher incidence or more extensive development of aneuploidy in paragangliomas.

We wish to thank Mrs N.J. Kuipers-Dijkshoorn for her expert contribution to the flow-cytometry studies, the Nijbakker-Morra foundation for financial support of our FACSCAN, and J.H.M. Frijns for editorial assistance.

### References

ANNIKO, M., TRIBUKAIT, B. & WERSÄLL, J. (1984). DNA ploidy and cell phase in human pituitary tumors. *Cancer*, **53**, 1708.

ARIAS-STELLA, J. & VALCARCEL, J. (1973). Chief cell hyperplasia in the human carotid body at high altitudes. *Human Pathol.*, **7**, 361.

BARNES, L. & TAYLOR, S.R. (1990). Carotid body paragangliomas (a clinicopathologic and DNA analysis of 13 tumors). *Arch. Otolaryngol. Head Neck Surg.*, **116**, 447.

BATSAKIS, J.G. (1982). *Tumours of the Head and Neck. Clinical and Pathological Considerations.* Second edition, p. 369. Williams & Wilkins: Baltimore.

BRACKMANN, D.E. (1988). The need for skull-base surgery in paragangliomas. In *Dilemmas in Otorhino-laryngology*, Harrison, D.F.N. (ed.) p. 91. Churchill Livingstone: London.

CHEDID, A. & JAO, W. (1974). Hereditary tumours of the carotid bodies and chronic obstructure pulmonary disease. *Cancer*, **33**, 1635.

CORNELISSE, C.J., VAN DE VELDE, C.J.H., CASPERS, R.J.C., MOOLENAAR, A.J. & HERMANS, J. (1987). DNA ploidy and survival in breast cancer patients. *Cytometry*, **8**, 225.

CORNELISSE, C.J. & TANKE, H.J. (1990). Flow cytometry applied to cytopathology. In *Comprehensive Cytopathology*, (in press). Bibbo, M. (ed.) W.B. Saunders Company.

GRIMLEY, P.M. & GLENNER, G.G. (1967). Histology and ultrastructure of carotid body paragangliomas. *Cancer*, **20**, 1473.

HEDLEY, D.W., FRIEDLANDER, M.L., TAYLOR, I.W., RUGG, A. & MUSGROVE, E.A. (1983). Method for analysis of cellular DNA content of paraffin-embedded pathological material using flow cytometry. *J. Histochem. Cytochem.*, **31**, 1333.

JOENSUU, H. & KLEMI, P.J. (1988). DNA aneuploidy in adenomas of endocrine organs. *Am. J. Pathol.*, **132/1**, 145.

KOSS, L.G., CZERNIAK, B., HERZ, F. & WERSTO, R.P. (1989). Flow cytometric measurements of DNA and other cell components in human tumours. *Hum. Patho.*, **20**, 528.

LACK, E.E., CUBILLA, A.L. & WOODRUFF, J.M. (1979). Paragangliomas of the head and neck region. A pathologic study of tumours from 71 patients. *Human Pathol.*, **10**, 191.

MERKEL, D.E. & MCGUIRE, W.L. (1990). Ploidy, proliferative activity and prognosis DNA flow cytometry in solid tumours. *Cancer*, **65**, 1194.

PAUW, K.H., KRENNING, E.P., VAN URK, H. & 7 others (1989). Scintigraphy of glomus tumours with $^{123}$I-labelled tyr-3-octreotide, a synthetic somatostatin (SMS) analogue. Proceedings Politzer Society.

REUBI, J.G. & TORHORST, I. (1989). The relationship between somatostatin, epidermal growth factor, and steroid hormone receptors in breast cancer. *Cancer*, **64**, 1254.

RODENBURG, C.J., CORNELISSE, C.J., HEINTZ, P.A.M., HERMANS, J. & FLEUREN, G.J. (1987). Tumour ploidy as a major prognostic factor in advanced ovarian cancer. *Cancer*, **59**, 317.

ROSENWASSER, H. (1975). Long-term results of therapy of glomus jugulare tumours. *Arch. Otolaryngol.*, **97**, 49.

SALDANA, M.J., SALEM, L.E. & TRAVEZAN, R. (1973). High altitude hypoxia and chemodectomas. *Human Pathol.*, **4**, 251.

SCHELFHOUT, L.J.D.M., CORNELISSE, C.J., GOSLINGS, B.M. & 4 others (1990). Frequency and degree of aneuploidy in benign and malignant thyroid neoplasms. *Int. J. Cancer*, **45**, 16.

SCHWARTZ, D., BANNER, B., ROSEMAN, D.L. & COON, J.S. (1986). Origin of multiple 'primary' colon carcinomas. A retrospective flow cytometric study. *Cancer*, **58**, 2082.

SCIALLERO, S., BRUNO, S., DI VINCI, A., GEIDO, E., ASTE, H. & GIARETTI, W. (1988). Flow cytometric DNA ploidy in colorectal adenomas and family history of colorectal cancer. *Cancer*, **61**, 114.

SHACKNEY, S.E., SMITH, C.A., MILLER, B.W. & 5 others (1989). Model for genetic evolution in human solid tumours. *Cancer Res.*, **49**, 3344.

SMIT, V.T.H.B.M., FLEUREN, G.J., HOUWELINGEN, J.C. VAN., ZEGVELD, S.T., KUIPERS-DIJKSHOORN, N.J. & CORNELISSE, C.J. (1990). Flow cytometric DNA ploidy analysis of synchronously occurring multiple malignancies of the female genital tract. *Cancer*, (in press).

STILLER, D., KATENKAMP, D. & KUTTNER, K. (1975). Jugular body tumours: Hyperplasia or true neoplasms? *Virchow's Arch.*, **365**, 163.

VAN GILS, A.P.G., VAN DER MEY, A.G.L., HOOGMA, R.P.L.M. & 4 others (1990). I-123 Metaiodobenzylguanidine scintigraphy in patients with chemodectoma of the head and neck region. *J. Nucl. Med.*, **31**, 1147.

VAN DEN INGH, H.F., GRIFFIOEN, G. & CORNELISSE, C.J. (1985). Flow cytometric detection of aneuploidy in colorectal adenomas. *Cancer Res.*, **45**, 3392.

VAN DER MEY, A.G.L., MAASWINKEL-MOOY, P.D., CORNELISSE, C.J., SCHMIDT, P.H. & VAN DE KAMP, J.J.P. (1989). Genomic imprinting in hereditary paragangliomas: evidence for new genetic theory. *Lancet*, **i**, 1291.

VINDELOV, L.L., CHRISTENSEN, I.J. & NISSEN, N.I. (1983*b*). A detergent trypsin method for the preparation of nuclei for flow cytometric DNA analysis. *Cytometry*, **3**, 323.